# The complete chloroplast genome of the Chinese banyan tree *Ficus microcarpa*

**DOI:** 10.1080/23802359.2021.1993097

**Published:** 2022-02-28

**Authors:** Enwen Lin, Zhenyang Liao, Xiuming Xu, Xingtan Zhang, Jingping Fang

**Affiliations:** a College of Life Sciences, Fujian Normal University, Fuzhou, China; b Center for Genomics and Biotechnology, Fujian Agriculture and Forestry University, Fuzhou, China; c Key Laboratory of the Ministry of Education for Coastal and Wetland Ecosystems, College of the Environment and Ecology, Xiamen University, Xiamen, China

**Keywords:** Ficus, PacBio, chloroplast genome, phylogenomic tree, Moraceae

## Abstract

Banyan tree or *Ficus microcarpa* is a large perennial plant with extraordinary aerial roots from the Moraceae family. In this study, the complete chloroplast genome sequence of *F. microcarpa* was assembled using PacBio data. The chloroplast genome size is 141,611 bp, consisting of a large single-copy (LSC) region and a small single-copy (SSC) region of 101,835 bp and 9,676 bp, respectively, which are separated by a pair of 15,050 bp inverted repeat (IR) regions. The genome includes 74 protein-coding genes, 43 tRNA genes, and 8 rRNA genes. A phylogenetic tree reconstructed by 25 complete chloroplast genomes reveals that *F. microcarpa* is mostly related to *Ficus racemosa.*

Banyan trees, best noted for their numerous aerial roots and large roofs, are large perennial evergreen plants with great ecological and cultural values. Nowadays Banyan trees are wildly grown in tropical and subtropical regions worldwide and used for urban landscaping. The Chinese Banyan *Ficus microcarpa* taxonomically belongs to the genus *Ficus*, which is the largest genus with ∼800 species under the Moraceae family (Berg 1989) and an ancient genus for about 75 million years old (Cruaud et al. 2012). Much attention has been drawn to the genetics and genomics of *Ficus* species owing to their special properties such as enclosed inflorescence, fig-wasp obligate mutualism and a hemi-epiphytic habit. The recent banyan genome project unveiled a *F. microcarpa* genome assembly of 426 Mb with 94.4% gene BUSCO completeness (Zhang et al. 2020). Chloroplast (cp) is an indispensable organelle for photosynthetic organisms, in which photosynthesis and other essential biochemical processes take place. The tiny size and simple structure of cp genomes make it an ideal model for taxonomic classification, phylogeny construction, and comparative genomic analysis in plants. However, no report has been published for the cp genomic characterization of *F. microcarpa* so far. The PacBio sequencing strategy combined with the correction of Next Generation Sequencing (NGS) data was proved to be a highly effective and accurate way to assemble high-quality circular cp genomes (Chen et al. 2015, Fang et al. 2020). Here, the complete *F. microcarpa* cp genome was assembled based on 36.87 Gb PacBio data and 52.6 Gb Illumina data from the banyan genome project (Zhang et al. 2020). We also collected a set of plastome data to resolve the evolutionary relationships within *Ficus*.

Young leaves of *F. microcarpa* were collected from Fujian Agriculture and Forestry University (FAFU), Fuzhou, China (119. 232124 E, 26.105562 N). The specimens were deposited in the laboratory of the Haixia Institute of Science and Technology, FAFU with specimen code FM20171201 (Mr. Lin, linenwe@126.com). For PacBio sequencing, 20-kb SMRTbell^TM^ libraries were prepared following the manufacturer’s protocol. A total of 30 Single-Molecule Real-Time (SMRT) cells were sequenced on the PacBio RSII system with P6-C4 chemistry. Before assembling a cp genome, potential chloroplast reads were extracted from the pool of PacBio reads using BLAST (Altschul et al. 1990) searches against the plastid reference database (https://ftp.ncbi.nlm.nih.gov/refseq/release/plastid/). All extracted PacBio reads were used to perform self-correction and *de novo* assembly of cp genome using CANU version 2.1 with default parameters (Kerdelhue et al. 2017), followed by error correction using the Quiver algorithm (Chin et al. 2013) and Pilon program (Walker et al. 2014). The ‘check_circularity.pl’ script provided by the spray package was used to check if the cp assembly has overlapping ends. Geseq (Tillich et al. 2017) was used to annotate the complete cp genome. The final cp genome of *F. microcarpa* was assembled into a circular-mapping molecule of 141,611 bp in length with an overall GC content of 36.83%, which has been deposited in GenBank with the accession number MW887640. This cp genome has a common quadripartite structure, comprising a large single-copy (LSC) region of 101,835 bp, a small single-copy (SSC) region of 9,676 bp, and two copies of inverted-repeat regions (IRa and IRb) of 15,050 bp. A to

tal of 125 genes were annotated, including 74 protein-coding genes, 8 ribosomal RNA (rRNA) genes, and 43 transfer RNA (tRNA) genes. The Tandem Repeat Finder (TRF) program (Benson 1999) identified 31 tandem repeats (TRs). The TR regions measure 1,387 bp (0.98%) in length with an extremely high AT content of 84.0%. The repeat units range in size from 26 bp to 89 bp and were present in 2–20 copies. The microsatellites (SSRs) distribution in this cp genome was analyzed based on MISA-web (Beier et al. 2017). The total number of SSRs is 54 with an average density of 381.33 SSRs/Mb. The repeat lengths range in size from 10 to 14 bp, all belonging to class II SSRs. Overall, the base composition of the SSR motifs showed a strong bias to AT-rich in this cp genome.

To confirm the phylogenetic position of *F. microcarpa*, complete cp genome sequences in *Ficus* genus and several closely related genera in Moraceae were used as data sets to reconstruct a phylogenetic tree. Sequences of twenty-four complete cp genomes were downloaded from NCBI GenBank. The single-copy orthologues of all protein sequences were aligned by MAFFT v7 (Koren et al. 2017) and the maximum-likelihood tree was constructed by raxmlHPC-PTHREADS-SSE3 (v8.2.12) ( Alexandros 2014) software with PROTGAMMAJTT model and 1000 bootstrap replicates (Figure 1). The *Debregesia orientails* and *Cecropia pachystachya* were used as outgroups. The phylogenetic tree reveals that all fifteen analyzed *Ficus* species form a monophyletic group and *F. microcarpa* in the current study is closely related to *Ficus racemosa*. The complete cp genome of *F. microcarpa* could provide fundamental data for further genetic and evolutionary studies on Moraceae species.

**Figure 1.**
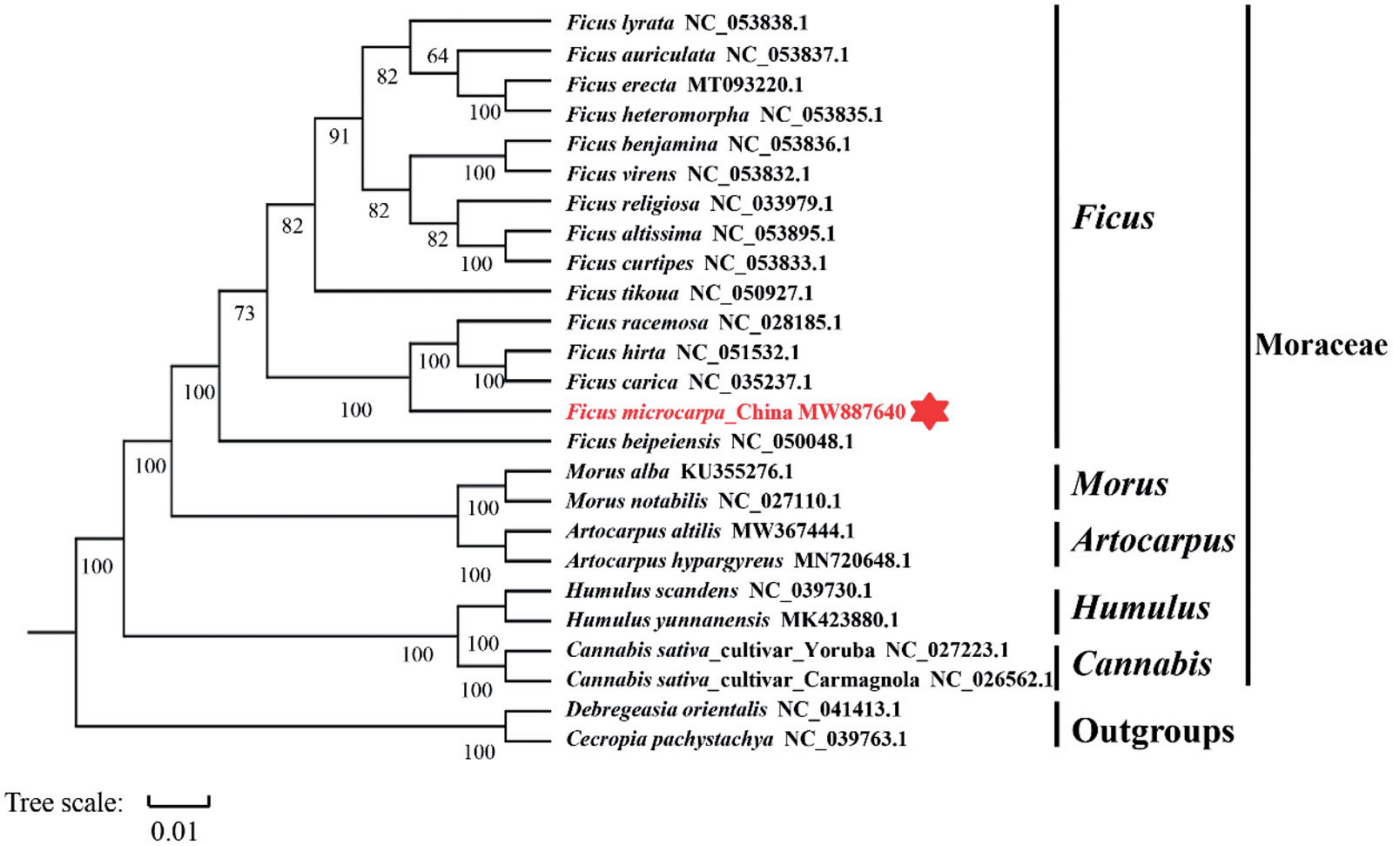
The Maximum likelihood (ML) phylogenetic tree is based on complete chloroplast genomes of *F. microcarpa* and other 24 species.

## Data Availability

The data that support the findings in this study are openly available. The complete chloroplast genome sequence of *F. microcarpa* has been deposited in Genbank with accession number MW887640 (https://www.ncbi.nlm.nih.gov/nuccore/MW887640.1/). The associated BioProject, SRA, and BioSample accession numbers are PRJCA002187, SRP298651, and SAMEA5192484, respectively.
